# A Novel Review of Homocysteine and Pregnancy Complications

**DOI:** 10.1155/2021/6652231

**Published:** 2021-05-06

**Authors:** Chuce Dai, Yiming Fei, Jianming Li, Yang Shi, Xiuhua Yang

**Affiliations:** ^1^China Medical University, Shenyang, Liaoning, China; ^2^Department of Obstetrics, The First Hospital of China Medical University, Shenyang, Liaoning, China

## Abstract

Homocysteine (Hct) is a substance produced in the metabolism of methionine. It is an essential type of amino acid gained from the daily diet. Methylenetetrahydrofolate reductase (MTHFR) gene mutation is related to elevated total homocysteine (tHct) expressions, in particular, among women with low folate intake. Hyperhomocysteinemia (HHct) is caused by numerous factors, such as genetic defects, lack of folic acid, vitamin B_6_ and B_12_ deficiency, hypothyroidism, drugs, aging, and renal dysfunction. Increased Hct in peripheral blood may lead to vascular illnesses, coronary artery dysfunction, atherosclerotic changes, and embolic diseases. Compared to nonpregnant women, the Hct level is lower in normal pregnancies. Recent studies have reported that HHct was associated with numerous pregnancy complications, including recurrent pregnancy loss (RPL), preeclampsia (PE), preterm delivery, placental abruption, fetal growth restriction (FGR), and gestational diabetes mellitus (GDM). Besides, it was discovered that neonatal birth weight and maternal Hct levels were negatively correlated. However, a number of these findings lack consistency. In this review, we summarized the metabolic process of Hct in the human body, the levels of Hct in different stages of normal pregnancy reported in previous studies, and the relationship between Hct and pregnancy complications. The work done is helpful for obstetricians to improve the likelihood of a positive outcome during pregnancy complications. Reducing the Hct level with a high dosage of folic acid supplements during the next pregnancy could be helpful for females who have suffered pregnancy complications due to HHct.

## 1. Introduction

**Figure 1:**
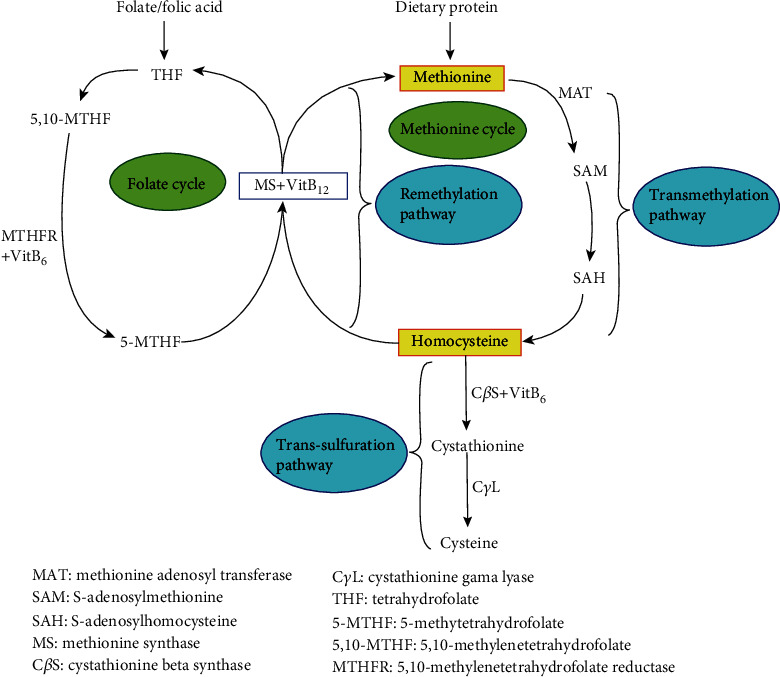
The metabolization of homocysteine (Hct) in the body. Hct is formed by transmethylation of methionine via S-adenosylmethionine (SAM) and S-adenosylhomocysteine (SAH) by methionine adenosyltransferase (MAT) (transmethylation pathway). Hct can be remethylated to methionine (remethylation pathway) or transsulfurated to cystathionine and cysteine (transsulfuration pathway). The transsulfuration pathway requires the catalysis of vitamin B_6_-dependent cystathionine beta-synthase (C*β*S). The remethylation of Hct to methionine is catalyzed by the vitamin B_12_-dependent methionine synthase (MS). Tetrahydrofolate (THF) is recycled to form 5-methyltetrahydrofolate (5-MTHF), catalyzed by 5,10-methylenetetrahydrofolate reductase (MTHFR). Folic acid can be used as a primary substance to produce 5-MTHF, and 5-MTHF could produce methionine and Hct. Thus, the reduction of folic acid could lead to hyperhomocysteinemia (HHct).

Homocysteine (Hct) is a substance produced in the metabolism of methionine. It is an essential type of amino acid gained from the daily diet. A proportion of Hct binds to serine and creates cystathionine, which is an enzyme-regulated response with the aid of cystathionine beta-synthase (C*β*S) and the accessory factor vitamin B_6_. However, most Hct is remethylated to form methionine. It is a procedure that needs a practical effect of a few enzymes. Methionine synthase (MS) increments a methyl unit to Hct combining with vitamin B_12_ and uses 5-methyltetrahydrofolate (5-MTHF) as a conjoint substrate (Figure 1). 5-MTHF synthesis requires plenty of reduced folate and an appropriate methylenetetrahydrofolate reductase (MTHFR) effect. MTHFR gene mutation is related to elevated total homocysteine (tHct) expressions, in particular, among women with low folate intake [1].

MTHFR gene polymorphism is a leading cause of hyperhomocysteinemia (HHct), and ten to twenty percent are homozygous for MTHFR 677C>T or 1298A>C [2]. The MTHFR gene is located at chromosome 1p36.3 and comprises 11 exons. Up to date, an abundance of DNA sequence changes have been discovered in this gene; 677CT and 1298AC are the best-known ones. 677CT transforms alanine at amino acid 222 into valine, resulting in a reduction of gene activity. Compared with the CC genotype, the TT genotype only exhibited 10-20% of gene activity. Meanwhile, the CT genotype had 60% of gene function [1]. 1298AC replaces glutamate at amino acid 249 with alanine. Consequently, the activity and thermal stability of the MTHFR gene are affected [1, 3]. Vitamins B_6_ and B_12_ and folate acid play vital roles in influencing the functionality of Hct. Reportedly, folic acid improved the efficacy of MS, which was dependent on vitamin B_12_ to convert Hct to methionine [4]. Also, Hct is related to lipid metabolism, even although the precise underlining mechanism is not clear. Studies have reported that HHct leads to hypomethylation and fat collection among tissues [5]. Thus, poor dietary balance and a sedentary lifestyle could result in HHct and obesity.

Numerous factors cause HHct, including genetic defects, lack of folic acid, vitamin B_6_ and B_12_ deficiency, hypothyroidism, drugs, aging, and renal dysfunction [6]. HHct corresponds to greater than 15 mmol/L on an empty stomach or more than 51 mmol/L after methionine administration in nonpregnant females. And both data are over the 97.5th percentile [7]. Increased Hct in peripheral blood may lead to vascular illnesses [8], coronary artery dysfunction [9], atherosclerotic changes [8, 10], and embolic diseases [11]. The production of nitric oxide (NO) and prostacyclin could be the factors that make HHct lead to thrombosis, which triggers a coagulation process, eventually leading to endothelial injury [12]. Studies have suggested that Hct can reduce the utilization activity of NO, increase oxidative stress, promote the proliferation of vascular smooth cells, and increase the inflammatory cytokines produced by vascular endothelial cells [13].

Compared to nonpregnant women, the Hct level is lower in normal pregnancies. The lower Hct level is due to the hemodilution caused by the increased blood volume and elevated glomerular filtration rate, and the fetus could absorb a proportion of Hct during pregnancy [14]. The Hct level reduces during early pregnancy; it reaches its lowest value during the second trimester; following this, it steadily increases during late pregnancy until it reaches the level of early pregnancy [15]. The normal values of Hct during pregnancy are as follows: 3.9–7.3 mmol/L before 16 gestational weeks, 3.5–5.3 mmol/L between 20 and 24 gestational weeks, and 3.3–7.5 mmol/L after 36 gestational weeks [15].  Table 1 shows the Hct levels in different stages of normal pregnancy reported in previous studies. Even though the value of Hct in late pregnancy is less than that before pregnancy, pregnant women are more susceptible to be impaired by HHct [30]. HHct is conducive to the production of hydrogen peroxide and superoxide free radicals. These adversities will lead to oxidative injuring of endothelial cells [31], diminished blood vessels in villi, and lower blood circulation at the maternal-fetal interface and ultimately lead to poor maternal and neonatal endings. Additionally, HHct promotes cell apoptosis, which induces trophoblast dysfunction [32]. HHct reduces NO released by endothelial cells, and it induces platelet accumulation and promotes thrombosis [33], which in turn affects placental perfusion [34].

According to recent studies, HHct was associated with numerous pregnancy complications, including recurrent pregnancy loss (RPL) [34–39], preeclampsia (PE) [40, 41], preterm delivery [42, 43], placental abruption [3, 44, 45], fetal growth restriction (FGR) [46–48], and gestational diabetes mellitus (GDM) [49, 50]. However, a number of these findings lack consistency [16, 36, 51–61]. Clinically, many practitioners ignore detecting Hct, mainly because they lack an understanding of its roles in pregnancy. This review is aimed at addressing the research progress of Hct and related pregnancy complications; the work done is helpful for obstetricians to improve the likelihood of a positive outcome during pregnancy complications. Two independent researchers searched the articles in PubMed with the following medical subject headings (MeSHs): “homocysteine,” “recurrent pregnancy loss,” “recurrent miscarriages,” “preeclampsia,” “placental abruption,” “fetal growth restriction,” “gestational diabetes mellitus,” or “pregnancy.” All articles were published in English from January 1998 to November 2020. We ruled out the letters.

## 2. Hct and RPL

RPL refers to three or more abortions by the European Society of Human Reproduction and Embryology (ESHRE) [62]; however, the American Society for Reproductive Medicine regards it as two or more spontaneous abortions [63]. The morbidity of RPL is 5% among couples of childbearing age [64]. Many reasons may lead to RPL, such as genetic issues [65], chromosomal abnormalities, anatomical deformities, endocrine problems, and immune diseases [66].

Hereditary thrombophilic deficiencies and increased tHct expressions, or a combination of these two pathogenic problems in RPL, lead to genetic susceptibility to venous thrombosis [35]. A third of RPL patients had HHct [35]. Since increased Hct can cause endothelial cell damage, it could harm the placental blood vessels, leading to RPL. Studies have found that elevated inflammatory cytokines, including tumor necrosis factor- (TNF-) *α* and HHct, are associated with insulin resistance (IR) and endocrine dysfunctions [67, 68]. It is likely for IR to result in an early abortion; it reduces the blood flow of endothelial cells and destroys the completeness of blood vessels [68]. Reportedly, high levels of Hct on fasting or after a meal and decreased folate levels are both high-risk factors for RPL [69].

Evidently, compared to women with MTHFR 677CC or 677CT genotypes, females with the MTHFR 677TT genotype exhibited lower vitamin D expressions, higher Hct levels, and increased natural killer (NK) cell cytotoxicities [37]. Vitamin D deficiency could elevate the Hct level by reducing the enzyme C*β*S, which occurs in the metabolism of Hct. C*β*S is a target for the vitamin D receptor; thus, vitamin D influences the Hct level. In females with the MTHFR 677TT genotype, both HHct and decreased expressions of 25(OH)VD could be responsible for RPL [37]. Reportedly, the C allele has a specific antagonistic effect on the increased level of Hct. However, the T allele could elevate the level of Hct and be a risk factor for RPL [70]. The MTHFR 677CT genotype can raise the morbidity of RPL by two to three times [38]. A recently conducted study showed that MTHFR 677CT and MTRR 66AG gene mutations result in harmful effects on serum Hct expressions [71]. However, one study suggested that there were no significant differences in Hct expressions and red blood cell (RBC), folate, and vitamin B_12_ expressions in terms of the appearance of homozygous and heterozygous MTHFR gene mutations between 60 unexplained RPL and 30 normal pregnant women with at least one neonate [36]. In the Tunisian population, 350 RPL women and 200 normal controls were tested for tHct levels, and the results of the study did not find any significant correlation between tHct and RPL [34]. The negative association implies that RPL has a diverse etiology, which involves numerous genetic pathways. During early pregnancy, the invasive capacity of the trophoblast determines whether the blood flow at the maternal-fetal interface is sufficient to promote fetal development; however, it is different from the blood circulation in other organs. Maternal gene mutations result in renal or cardiac embolic diseases; however, they may not have identical harmful effects on placental circulation during the first trimester of pregnancy [34]. There are 7 articles which matched the MeSHs for the RPL section: 5 articles supported and 2 articles rejected the association between Hct levels and RPL (Table 2). Inconsistent results are attributable to varying study populations and different research designs.

A positive correlation exists between the MTHFR 677CT genotype and the lipoprotein expressions in RPL [78]. Due to the positive association between MTHFR 677CT and 1298AC and serum Hct expression [79, 80] and the positive functionality of Hct on lipid production [79], the authors proposed that the MTHFR 677CT and 1298AC genotypes and elevated Hct collectively increased the abnormal lipid metabolism in RPL patients.

## 3. Hct and PE

In general, PE is characterized as hypertension (≥140/90 mmHg) accompanied by proteinuria, which is found after 20 gestational weeks [81]. It is a disease involving multiple organs, including the nervous system, kidney, liver, and circulatory system. Although its etiology is still unclear, endothelial dysfunction, inflammation, and immune abnormalities are several possible causes [82].

In this section, 16 related articles were identified: 11 articles supported and 5 articles opposed the relationship between Hct levels and PE. Recent studies have reported that the expression levels of Hct in PE women were significantly higher than those in normotensive pregnant women [74–76] (Table 2). However, a significant difference between PE patients and nonpregnant women was not found [72], implying that HHct in PE could be due to a change in the blood volume rather than MTHFR gene mutation [72]. Recently, a case-control study found that following the adjustment of confounding factors in logistic regression models, the OR value for PE was 1.16 (95% CI: 1.05-1.27) for 1SD enhancement in Hct [77]. The study identified that a high maternal serum Hct level was a risk factor for PE [77]. Statistically, Hct is directly associated with the seriousness of hypertension during pregnancy [76]. Despite the levels of folic acid and vitamin B_12_ in PE patients being identical to those in normal pregnant women, the expression level of Hct in the PE group was still higher [73]. The elevated level of Hct was negatively associated with the levels of docosahexaenoic acid (DHA), which was reduced in PE [73]. The reduction of antioxidants and excessive oxidative stress lead to a reduction of DHA, which is the main cause of PE [83]. The elevated level of plasma Hct in pregnant women could result in decreased erythrocyte phospholipid DHA in newborns, which would affect the development of the neonatal nervous system [84].

The imbalance of the Hct-asymmetric dimethylarginine- (ADMA-) NO pathway could also be related to PE, and it is a critical sign of the seriousness of PE [40]. Compared to healthy pregnant women, both Hct and ADMA in serum of early-onset PE and late-onset PE were considerably higher [41]. Meanwhile, as clinical symptoms occurred, Hct and ADMA increased in patients with early-onset PE [41]. According to this study, reducing the elevated Hct expressions may benefit PE with vascular disorders. Discussing the impact of minimizing the levels of Hct and ADMA in PE through a large-scale prospective study is needed in future analysis. Reportedly, experiments involving animals showed that HHct did not cause PE-related symptoms in pregnant rats; however, HHct could inhibit fetal development and increase the likelihood of intrauterine fetal fatalities [85]. Compared to the group of control rats, the birth weights were significantly less in methionine-fed rats, and the proportion of fetal death was 15% higher [85].

Several empirical studies are aimed at exploring whether Hct is utilizable for predicting PE in low-risk pregnant women [16, 58, 86–88]. In retrospect, Hct was detected in reserved blood specimens during the first trimester [87, 88] or the second trimester of pregnancy [16, 58, 86]. Resultantly, the Hct expressions were clearly related to PE in three studies [86–88], yet it was irrelevant in the other two [16, 58]. The reason for the high Hct level in early pregnancy could be due to the impairment of vascular endothelial cells in the first trimester. As the pregnancy processes, this injury aggravates placental ischemia, eventually leading to PE [88]. Moreover, a study measured Hct in the second trimester in pregnant women with chronic hypertension, and the tests were used for predicting whether PE occurred in the third trimester [59]. Each participant was administered folic acid orally [59]. The sensitivity and specificity of Hct in predicting PE were 13% and 95.1%, respectively [59]. Therefore, the expression level of Hct in the second trimester does not help predict PE in pregnant women with chronic hypertension.

Another study tested the serum Hct in women with normotensive (*n* = 1825) and hypertensive (*n* = 401) pregnancies [89]. The study discovered that in comparison to normal pregnancies, a record of pregnancy-induced hypertension (PIH) was related to a 4.5% higher serum Hct level (*P* < 0.05) and 1.6-fold elevated odds of displaying an elevated Hct [89]. Several years after the pregnancy, the Hct levels increased in females with a history of PIH. The Hct expression is a valuable indicator of cardiovascular diseases several years later.

## 4. Hct and Preterm Delivery

Premature delivery refers to the termination of pregnancy before 37 gestational weeks due to medical reasons or spontaneous labor. It is still a major obstetrical disease and stimulates extensive social concern. In China, the prevalence of preterm birth is 7.2%; comparatively, in the United States, it is 9.6% [90, 91]. Globally, there are around 15 million premature births annually [92]. There are numerous causes of preterm delivery, including genital malformations, systemic inflammation, and other pregnancy complications [93]. Lately, numerous studies have confirmed an association between mutations of folate metabolism-related genes and preterm delivery [94].

Since the impact of Hct on endothelial cells could result in vascular obstruction, HHct could be associated with preterm labor. We found 7 relevant articles: 6 articles supported and 1 article rejected the association between Hct levels and preterm delivery. A case-control study analyzed 277 preterm births and 444 full-term pregnancies [42]. The expression of Hct was remarkably advanced in patients with vascular lesions in the decidua; however, the prevalence of decidual vasculopathy showed no statistical significance with preterm delivery [42]. Therefore, similar to coronary artery disease [95], the increased Hct level could lead to or become a potential marker of placental vascular disease. It could cause or speed up the process by stimulating hormone secretion and inflammation formation or increasing the cellular gap links, thus causing premature delivery [42]. A similar study conducted in China included 300 preterm pregnant females together with their newborns and 300 normal-term controls [43]. It was revealed that the serum Hct levels were much higher in preterm infants than in normal neonates, and this finding conformed with the trend of Hct expression in maternal serum [43]. The reason may be that the newborn shares a connection with the mother through the umbilical cord. As a result, the expression level of a substance in the newborn's serum agrees with that of the mother. An identical pattern is sustained a day after delivery. The level of Hct in maternal serum is an effective predictor of premature delivery, and it can be regarded as a novel objective index to prevent and treat premature delivery [43]. In *in vitro* experiments on the myometrium obtained during cesarean section, they found that Hct could promote idiopathic contraction of the myometrium [96]. HHct can inhibit the activation of methyltransferase and make uterine contractions more frequent with a higher amplitude, which induces premature delivery [97].

The level of Hct was associated with the history of preterm birth [98]. Compared with patients with the lower quartile of plasma tHct, patients with the upper quartile exhibited a 38% higher chance of going through premature birth [98]. Accordingly, the study suggested a significant correlation between the history of preterm birth and the maternal plasma Hct levels attained a few years postdelivery. Another study indicated that total cysteine (tCys) levels had a remarkable association with a history of preterm birth [99]. The tHct levels were associated with preterm birth only when tCys levels increased [99]. The rise in tCys characterized the deterioration of renal function, resulting in increased expressions of both tHct and tCys [100]. Also, another study analyzed the relationship between the preconception Hct levels and the risk of preterm birth in the index pregnancy. It included 29 preterm births and 405 normal-term pregnancies [101]. The Hct levels during nonfasting conditions were measured [101]. According to the results, if the Hct level exceeded 12.4 *μ*mol/L before pregnancy, the prevalence of preterm birth was four times higher (*P* < 0.05) [101]. The research found a significant correlation between Hct before conception and preterm birth among the Chinese population.

A systemic review that examined manuscripts published between January 1980 and May 2014 was conducted [102]. Three of the included manuscripts discussed the association between Hct and spontaneous preterm birth. Two articles suggested that the elevated levels of Hct in the second trimester and during delivery were both linked to premature delivery [42, 103]. However, one article revealed that Hct expression during late pregnancy was not related to preterm delivery [51]. The results of these studies further help practitioners understand the etiology of preterm birth. Besides, the role of Hct in preterm labor is attributable to vascular injury caused by HHct on endothelial cells [104].

## 5. Hct and Placental Abruption

Placental abruption refers to the rupture of uterine spiral arteries that causes partial or total placental segregation before delivery. The diagnosis of placental abruption depends on clinical symptoms or signs. Patients with placental abruption may have obvious abdominal pain and vaginal bleeding. The diagnosis is confirmed by pathological examination of placental tissue after delivery [45].

A total of 5 articles matched the MeSHs for this section: 4 articles supported and 1 article rejected the association between Hct levels and placental abruption. An empirical study among the Dutch population found that HHct appeared in 26 of 84 patients (31%) with placental vascular disease (including placental abruption) during postpartum (6-49 months), compared with 4 of 46 normal pregnancies (9%) (*P* < 0.05) [44]. Besides, the levels of fasting Hct in patients with placental abruption were noticeably higher than the levels in normal pregnancies [44]. A retrospective case-control study involved 77 placental abruption patients and 77 normal pregnancies with no signs or symptoms of prenatal bleeding [45]. According to the univariate analysis, increased Hct expression (OR: 45.55; 95% CI: 7.05–458.93) posed a significant risk factor for placental abruption [45]. Moreover, another research involved 46 females with placental abruption (experimental group) and 100 healthy pregnant females (control group) who had one normal birth at least [3]. Blood was drawn from all pregnant women during the third trimester [3]. Accordingly, the mean plasma Hct level in the experimental group was significantly higher compared to the control group (9.59 ± 2.35 vs. 6.07 ± 2.17 *μ*mol/L, *P* < 0.05) [3]. The study suggested that high Hct might predict placental abruption [3]. A systematic review containing 18 studies implied that HHct, in both the fasting and after-methionine situations, was significantly prevalent in patients with placental abruption [105]. However, the quality of the tests in this review is at a common level, and the selection of control groups is not strict enough; as a result, there may be errors in different laboratory measurements. Extensive studies are necessary to determine the consistency of these results. Reliable results will guide the diagnosis and treatment of placental abruption.

Another empirical study with a large sample size included 5883 women (40-42 years old) who had a total of 14,492 pregnancies [98]. The outcomes of the pregnancies and complications were retrospectively analyzed [98]. The comparison of the upper quartile of plasma tHct and lower quartile did not show any significant association between tHct and placental abruption [98]. However, the comparison of patients with higher tHct levels (more than 15 mmol/L) with those having lower quartile levels showed that the prevalence of placental abruption was statistically higher in the former group (*P* < 0.05) [98]. Moreover, another study involved 7587 subjects [52]. The authors performed multivariable logistic regression calculations using Hct as the first persistent variable. After regulating gestational weeks during blood drawing and general characteristics of subjects, the authors revealed that elevated Hct was not associated with placental abruption [52]. The difference in experimental results may be related to the diverse definitions of HHct.

## 6. Hct and FGR

FGR is a condition where the fetus is unable to reach its growth proficiency. In clinical terms, FGR generally defines that the birth weight should be lower than the 10th percentile or 2 standard deviations of the average weight of the same gestational age or should not exceed 2500 g after 37 weeks of gestation [106]. The etiology of FGR is attributable to maternal factors, placental or umbilical cord problems, or abnormal fetal development [107].

We confirmed 10 related articles: 7 of them supported and 3 articles rejected the association between Hct expressions and FGR. High levels of Hct can impact the transfer of amino acids in the placenta, which could cause FGR [48]. The plausible mechanisms are as follows. Firstly, FGR is characterized by a reduction in the activity of placental carriers of neutral amino acids, such as Systems A and L [108]. Secondly, the absorption of placental amino acid is reduced in FGR [109]. HHct is more prevalent in FGR, and it can restrain the transfer of endogenous amino acids in the placenta. It is a critical process since essential amino acids are absolutely instrumental for the growth and development of the fetus. If maternal HHct can induce FGR by inhibiting amino acid transfer, it provides a nongenetic hypothesis for the inheritance of cardiovascular disease between mothers and infants. In *in vitro* experiments, moderate HHct could impede the transfer of amino acids and induce FGR [48].

A meta-analysis comprising 21,326 pregnant women from nineteen studies discovered that the incidence of FGR was higher when Hct exceeded the 95th percentile (OR 1.25; 95% CI: 1.09–1.44) [110]. Moreover, this review also suggested that when the Hct exceeded the 90th percentile, there was a 25% higher risk of delivering a baby with small for gestational age [110]. An empirical study based in Australia extracted the blood samples of 137 pregnant women during 18-20 weeks of gestation for Hct tests [111]. Analysis revealed that compared to normal pregnancies, the Hct levels in FGR patients were significantly higher [111]. The finding was also confirmed with low-income countries, such as South Asian nations [112]. Another empirical study collected the venous blood from 40 FGR women and 45 females who gave birth to a neonate with normal birth weight, and the collections were made within 24 hours after delivery [113]. It was confirmed that Hct expression in FGR women was considerably higher than that in normal pregnant females [113]. Therefore, the study suggested that the maternal plasma Hct level could serve as a predictive factor for FGR. Nonetheless, these findings are yet to be confirmed in other studies [16, 60, 61]. It could be due to isolated FGR, where endothelial cell damage is limited to uterine and placental blood circulation. In addition, negative results were found among African American women [16].

On the other hand, some authors believed that the risk of a woman having a neonate with FGR reduced with elevating tHct [114]. Then, the result was questioned [55]. Firstly, the authors pointed out that Hct tests were conducted within 48 hours after delivery [114]. So, the measurements fail to reflect the actual level of tHct at the time of delivery since it is likely that the Hct levels might have changed considerably 48 hours after delivery [55]. The optimal experimental design is to draw blood at different points of time for Hct testing, including preconception, early pregnancy, midpregnancy, late pregnancy, immediately after delivery, and one week after delivery. It is much more reliable to judge the statistical significance according to these values. Secondly, the absence of specific statistical data to support the conclusions that the results for tHct were significantly different between control neonates and experimental infants or between their mothers was questioned [55]. Therefore, detailed data is required to confirm the unexpected results [114] regarding the association between Hct and FGR.

## 7. Hct and GDM

GDM is a type of impaired glucose tolerance that occurs or is diagnosed for the first time during pregnancy [115]. Currently, there are two types of metabolic problems in GDM, namely, IR and *β* cell abnormalities. These fulfill a vital functionality in the etiology of GDM [116]. Some trials have demonstrated that the degree of IR is positively related to high Hct expression [117]. Therefore, some empirical studies have emphasized the role of plasma Hct in GDM.

Nine articles matched the MeSHs for this section: 5 articles favored and 4 articles rejected the association between Hct expressions and GDM. Reportedly, elevated levels of Hct are related to GDM in a few [49, 50, 118] but not all cross-sectional trials in Western countries [119–121]. Remarkably, serum Hct was elevated in patients with GDM and had a significant association with the 2-hour OGTT glucose value [49]. Compared to normal pregnancies, the serum Hct levels between 24 and 28 gestational weeks were elevated in GDM patients [50]. However, a study based in Poland tested serum Hct expressions among 60 GDM patients and 19 normal pregnant females [57]. The study did not find any difference in the Hct levels between the two groups [57]. Hct was unassociated with fasting blood glucose and glycosylated hemoglobin [57]. The negative results might be related to the small number of subjects of the control group (*n* = 19) in this study.

A case-control research was conducted in China; it contained 350 GDM women and 346 normal glucose tolerance (NGT) pregnant females [122]. Patients with GDM exhibited an elevated plasma tHct expression than NGT females (*P* < 0.05) [122]. The GDM risk was 1.79 times higher in patients with increased tHct (≥7.29 *μ*mol/L) relative to females with lower tHct expression (<5.75 *μ*mol/L) [122]. The association between Hct and GDM history has also been analyzed in an empirical study [123]. Patients with a history of GDM exhibited higher Hct levels [123]. A meta-analysis revealed an increased tHct expression in GDM women than normal pregnant patients [124]. However, the numbers of samples in each study in this meta-analysis were less than 250 cases. Therefore, expanding the study using a larger sample size is necessary to confirm the results.

Contrarily, studies have suggested that elevated levels of Hct were related to reduced fasting and 2-hour glucose numbers and decreased odds of GDM [120]. This finding does not conform with the theory that HHct leads to IR [125]. There have been contradictory findings in the correlation between Hct and GDM; it could be due to the cut-off values of Hct in various studies. The cut-off value of high Hct in this study [120] was significantly lower than the standard of commonly defined HHct (>16 mmol/L) [126] and was also lower than that related to GDM in other studies (≥6 mmol/L) [49, 50, 118]. Resultantly, this level of Hct failed to attain the degree of inducing metabolic changes.

## 8. Hct and Birth Weight

Nine articles matched the MeSHs for this part: 8 articles supported and 1 article rejected the association between Hct expressions and birth weight. Neonatal birth weight and maternal Hct levels were negatively correlated [127]. It could be due to the role of 1-C on nucleic acid synthesis and the effect of DNA methylation on fetal development since 1-C supplies the essential amino acid methionine for protein synthesis [128]. Numerous empirical studies involving animals have demonstrated that methyl obtained in the daily diet was crucial for the development of the fetus [129]. Additionally, HHct can cause harm to the vascular endothelium by diminishing NO and advancing inflammatory response and oxidative stress pathways, impacting the placental perfusion and function [130]. Other clinical trials have discovered a mild weight decline in neonates whose mothers have increased Hct expressions compared to females with normal birth weight newborns [131, 132]. Using multiple linear regression calculation, it was determined that the trend for a negative effect on birth weight of owning the highest maternal tHct level compared with the low-middle levels existed at preconception, 8, 20, 32 gestational weeks, and during delivery [133]. The tHct values of maternal and fetal cord blood were both inversely proportional to the birth weight [134]. HHct showed a strong connection with low birth weight before pregnancy or 10 years after childbirth [98]. If the maternal Hct value was located at the upper quartile (≥10.7 mol/L), the risk of giving birth to very low-birth weight infants increased by 2.07 times compared with the lower quartile [98]. One unit increase in Hct results in a loss of 33.3 grams in fetal weight [135]. Likewise, another study carried out among the Indian population obtained identical results [127]. In a prospective study, elevated Hct levels and low folic acid during early pregnancy were related to a lighter placenta and lower fetal weight [136].

In a retrospective research testing Hct, blood samples were drawn from 498 pregnant females during the first obstetric ultrasound scan. The outcomes of the study indicated that maternal Hct was unrelated to fetal weight [56]. Most importantly, a high Hct level was associated with maternal smoking and low economic status, and both these factors were related to low birth weight [56]. The analysis concluded that two confounding issues, namely, maternal smoking and low economic status, should be considered when analyzing the association between Hct and fetal birth weight [56].

## 9. Conclusions

The activity of Hct in normal pregnancy and pregnancy complications has been widely discussed. According to the literature, the Hct level during pregnancy is not constant; it is associated with geographic, cultural, and social characteristics of females. Evidently, elevated maternal Hct and polymorphisms of related genes in Hct metabolism are related to pregnancy complications. The advantage of comprehending the relationship between tHct and human pregnancy will undoubtedly be advanced by prospective trials, optimally, from preconception through all stages of pregnancy until the children's period. However, a clinical experiment of that sort is extremely resource-consuming. In the case where preconception and postpartum data are unavailable, the Hct levels can be gathered from early pregnancy at least. Reducing the Hct level with a high dosage of folic acid supplements during the next pregnancy could be helpful for females who have suffered pregnancy complications due to HHct.

## Figures and Tables

**Table 1:** Normal homocysteine (Hct) levels reported in the literature.

Authors	Population	Number of normal subjects	Hct levels (*μ*mol/L)		
			First trimester	Second trimester	Third trimester
Walker et al. [15]	Canadian	155	5.6 ± 1.6 (8-16 w)	4.3 ± 1.0 (20-28 w)	5.6 ± 2.3 (32-42 w)

Hogg et al. [16]	Italian	63	—	4.7 ± 1.4 (26 w)	5.3 ± 1.8 (37 w)

Bondevik et al. [17]	Nepalese	382	9.9 (9.1-10.6)	9.3 (8.6-10.1)	9.4 (8.5-10.3)

Murphy et al. [18]	Spanish	54	6.48 ± 1.30 (8 w)	5.22 ± 1.29 (20 w)	5.16 ± 1.32 (32 w)

Cotter et al. [19]	Irish	142	7.07 ± 1.5	—	—

Velzing-Aarts et al. [20]	West African	50	9.42 (95% RI: 5.50-16.12) (9 w)	7.28 (95% RI: 4.28-12.40) (16 w)	6.89 (95% RI: 3.93-12.06) (28 w)
				7.33 (95% RI: 4.25-12.64) (20 w)	7.17 (95% RI: 4.38-11.73) (32 w)
				7.11 (95% RI: 4.03-12.55) (24 w)	7.60 (95% RI: 4.46-12.96) (36 w)

Milman et al. [21]	Danish	406	—	6.4 (3.6-9.4) (18 w)	7.0 (4.0-9.7) (32 w)
					7.7 (5.2-12.0) (39 w)
					10.8 (6.8-19.3) (8 w after delivery)

Milman et al. [22]	Danish	434	—	6.06 (3.34-11.00) (18 w)	6.61 (3.93-11.10) (32 w)
					7.78 (4.72-12.81) (39 w)
					10.99 (5.85-20.64) (8 w after delivery)

Wallace et al. [23]	Seychelles	226	5.83 (4.03-10.38)	6.84 (4.33-12.93) (28 w)	12.4 (5.91-23.17) (delivery)

Hay et al. [24]	Nordic	364	—	4.7 (4.5-4.9) (17-19 w)	—

Hogeveen et al. [25]	Dutch	366	—	5.5 (4.5-6.7) (30-34 w)	—

Samuel et al. [26]	South Indian	360	9.22 (5.74-15.08)	—	—

Choi et al. [27]	Korean	278	10.6 (8.9-15.7) (5-13 w)	10.6 (8.2-13.9) (14-26 w)	10.2 (7.9-14.0) (27-40 w)

Maged et al. [28]	Egyptian	453	—	4.70 ± 2.08 (15-19 w)	—

Yang et al. [29]	Chinese	354	5.79-11.86	5.79-11.86	6.13-16.75

RI: reference interval.

**Table 2:** Case-control studies about homocysteine (Hct) in recurrent pregnancy loss (RPL) and preeclampsia (PE).

Authors	Experimental group	Control group	Results	Conclusions
RPL				
Nelen et al. [69]	123 RPL patients	104 normal women	Increased fasting Hct (≥18.3 mmol/L) and afterload Hct (≥61.5 mmol/L) were both associated with RPL.	Increased Hct was a risk factor for RPL.

Raziel et al. [35]	36 nonpregnant RPL patients	40 parous women	HHct was found in 31% of the RPL patients.	Patients with RPL were more likely to have HHct.

Zammiti et al. [34]	350 RPL patients	200 normal women	The tHct levels were similar between the two groups.	There was no association between the risk of RPL and tHct levels.

Creus et al. [36]	60 RPL patients	30 fertile females	There was no significant difference in the Hct levels between the two groups.	RPL was not associated with HHct.

Chakraborty et al. [68]	126 RPL patients with PCOS	117 normal women without PCOS	There was a significant difference in Hct expression between the experimental group and the control group (70.63% vs. 57.26%; *P* < 0.05).	HHct could increase the possibility of RPL.

Zarfeshan Fard et al. [70]	50 RPL patients	50 women having at least two normal pregnancies	The expression of Hct was higher in the experimental group (*P* = 0.002) compared to the control group. Increased Hct tended to be more common in women with the T allele.	The 677CT genotype may be a risk marker for abortion, and the C allele protected women from RPL.

Lin et al. [71]	403 RPL patients	342 normal females	The expression of Hct was higher in the experimental group relative to that in the control group.	MTHFR 677CT and MTRR 66AG gene mutations increased Hct expressions.

PE				
Raijmakers et al. [72]	20 PE patients	10 healthy nonpregnant females and 10 normotensive pregnant females	PE patients had higher Hct levels than normotensive pregnant women (13.3 vs. 8.4 mmol/L; *P* < 0.05).	Mild HHct may not be a risk marker for PE. HHct in PE was related to the changes of plasma volume instead of MTHFR gene mutation.

Mao et al. [40]	62 PE patients	30 normal pregnant women	Both the mild and severe PE patients exhibited higher Hct levels compared to controls.	The Hct-ADMA-NO pathway was involved in the cause of PE and associated with the severity of PE.

Kulkarni et al. [73]	49 PE patients	57 normotensive pregnant women	Despite there being no difference in folic acid and vitamin B_12_ levels between the two groups, the Hct levels were higher in the experimental group.	The reduction of DHA in PE was related to HHct.

Laskowska et al. [41]	62 early-onset PE and 53 late-onset PE patients	65 normotensive pregnant women	There were increased expressions of Hct in the serum of patients with PE, especially in the early-onset PE population.	The expression level of Hct was related to the severity of PE and could indicate early symptoms of PE.

Şanlıkan et al. [74]	30 severe PE and 24 mild PE patients	60 normal pregnant women	Hct levels in the control group were lower compared to those in the experimental group. A significant difference did not exist in Hct expression between the mild and severe PE patients.	Hct was significantly increased in PE patients, but it was not related to the severity of this disease.

Wadhwani et al. [75]	62 PE patients	126 normotensive pregnant women	PE patients had higher Hct levels compared with controls in the second trimester, third trimester, and during delivery.	Increased Hct expressions occurred in PE patients from the first trimester to delivery.

Maru et al. [76]	64 mild PE, 50 severe PE, and 32 eclampsia patients	68 healthy pregnant women	Hct greater than 8 mmol/L was associated with severe PE, and maternal complications tended to occur among these women.	Hct was usable as one of the predictors for PE.

Serrano et al. [77]	2978 PE patients	4096 normal pregnant females	The OR for PE was 1.16 (95% CI: 1.05-1.27) for 1SD enhancement in log-Hct.	HHct was one of the high-risk factors for PE.

HHct: hyperhomocysteinemia; PCOS: polycystic ovary syndrome; tHct: total homocysteine; ADMA: asymmetric dimethylarginine; NO: nitric oxide; DHA: docosahexaenoic acid.
